# Effectiveness of a quality improvement strategy with implementation of a specific visual tool to promote ICU early mobilization

**DOI:** 10.1038/s41598-022-21227-y

**Published:** 2022-10-13

**Authors:** Patricia Nery de Souza, Jessica Borges Kroth, Amanda dos Santos Ligero, Juliana Mesti Mendes, Ana Lígia Vasconcelos Maida, Laerte Pastore, Wellington Pereira Yamaguti

**Affiliations:** Hospital Sírio Libanês, São Paulo, Brazil

**Keywords:** Rehabilitation, Criticality, Disability

## Abstract

Early progressive mobilization is a safe strategy in the intensive care unit (ICU), however, it is still considered challenging by the inherent barriers and poor adherence to early mobilization protocol. The aim of this study was to evaluate the effectiveness of a quality improvement (QI) multifaceted strategy with implementation of a specific visual tool, the “mobility clock”, in reducing non-compliance with the institutional early mobilization (EM) protocol in adult ICUs. A single-center QI with a retrospective before-after comparison study was conducted using data from medical records and hospital electronic databases. Patients from different periods presented similar baseline characteristics. After the QI strategy, a decline in “non-compliance” with the protocol was observed compared to the previous period (10.11% vs. 26.97%, p < 0.004). The proportion of patients walking was significantly higher (49.44% vs. 29.21%, p < 0.006) and the ICU readmission rate was lower in the “after” period (2.25% vs. 11.24%; p = 0.017). The multifaceted strategy specifically designed considering institutional barriers was effective to increase out of bed mobilization, to reduce the “non-compliance” rate with the protocol and to achieve a higher level of mobility in adult ICUs of a tertiary hospital.

## Introduction

The continuous improvement in critical illness survival has led to increasing recognition of the long-term consequences of therapy in intensive care unit (ICU). This repercussion goes beyond the physical aspect and extends to social, cognitive, and mental health function^1^. At present, ABCDE bundle is considered a remarkable strategy to optimize ICU care recovery and outcomes^2^. Early progressive mobilization, represented as the “E” bundle component is a safe strategy to reduce ICU-acquired weakness (ICU-AW), which can cause a direct impairment of functional status. That condition can perpetuate after hospital discharge, affecting the quality of life and social reintegration^1–9^.

Although mobilization is considered essential and recommended to start immediately after physiologic stabilization, the number of patients in compliance with this recommendation that are mobilized out of bed is considered low and is less than predicted by known safety criteria and patient clinical condition^10,11^. In the ICU environment there are multifactorial barriers related to the structural context, process, culture, in addition to patient-related factors^7,10–15^. The improvement of patient’s mobility level must consider all those barriers present at the moment^14^ and, therefore, setting strategies to overcome them with a multidisciplinary approach towards early mobilization (EM) is recommended^14–24^.

The first EM protocol in our institution was developed in adult ICUs in 2011, and its use started in that same year. It contained four progressive intervention plans, the Medical Research Council (MRC) grading system and the Surgical Unit Optimal Mobilization Score (SOMS) were used, respectively, in patients who are alert and attentive and in those who were unable to follow commands^25^.

In 2018, ICU physical therapy team, based on the current literature, updated their EM knowledge. Due to that process, in our institution, EM came to be defined as activities with axial loading (sitting on the edge of the bed or greater levels of out of bed mobilization) as proposed by Harrold et al. in 2015 ^16^. After this update in the EM concept, we started to monitor the proportion of patients mobilized out of bed in the institutional indicator named as “verticalization” rate. Likewise, the proportion of patients who do not have contraindications but are not mobilized out of bed as prioritized in our EM protocol, came to be accounted by the indicator named as “non-compliance” rate. These indicators are collected monthly by a physical therapist through the patient’s medical record from the preceding 24 h, without the team’s previous knowledge in a one-day point-prevalence. The reasons not to mobilize as well, the barriers perceived by the physical therapist on duty are investigated at this moment. For any data uncertainties, the responsible physical therapist could question the health employees on duty.

The preliminary data analysis of this indicator demonstrated that our “verticalization” and the “non-compliance” rate was 36.3% and 24%, respectively. This result was considered as a nonconformity, since some level of out of bed mobilization was expected from those patients considering they had clinical conditions to be “verticalized” without any contraindications ^24^.

Based on the data provided from the institutional mobilization indicator and the multidisciplinary perception consolidated in a workshop, a multifaceted improvement strategy was proposed. The aim of our study was to evaluate the effectiveness of these strategies in reducing non-compliance with the institutional EM protocol in adult ICUs of a tertiary hospital.

## Methods

This study was a QI project that followed the revised Standards for Quality Improvement Reporting Excellence (SQUIRE2) guidelines^26^ (Supplementary).

### Ethics approval and consent to participate

This QI project received ethics approval from the research ethics committee of the Hospital Sírio Libanês/Sociedade Beneficiente de Senhoras (CEPesq/HSL), with a reference number: CEPesq HSL2019-84. This committee in addition to releasing the research protocol, waived the need of informed consent form since the study is a retrospective review of data from medical records and hospital electronic database. The methods were carried out in accordance with the relevant guidelines and regulation.

### Project design

**Figure 1:**
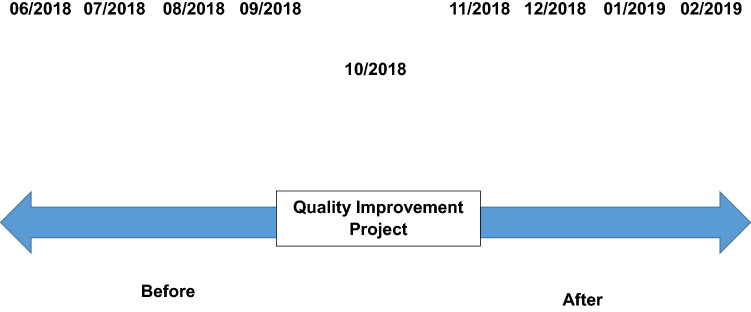
Study design.

This single-center QI with retrospective before-after comparison study was conducted using data from medical records and hospital electronic databases in 2019 at the Sírio-Libanês Hospital, São Paulo, Brazil (Fig. 1).

The primary study outcome was the “non- compliance” rate with the EM protocol. The mobility landmark achieved per period, a the lCU length of stay, the ICU and hospital mortality, and the ICU readmission rate were considered as secondary outcome parameters.

The data was collected based on the physiotherapy service “verticalization” rate. Consecutive patients who meet the inclusion and exclusion criteria of the study were part of the sample. Therefore, only patients aged ≥ 18 years who were included in the indicator screening, without a mobility contraindication was considered. Contraindication was defined as any hemodynamic, neurological, or respiratory instability; medical contraindications, medical indication to prioritize comfort measure, patient refusal; and patients admitted or discharged from the ICU on the day of collection.

The “before” period corresponded to the previous four months of the improvement strategy implementation in October 2018. The “after” period corresponded to the four months following the executed QI initiative. The data was obtained in a decreasing and growing way in the months until the sample size was achieved.

### Setting

During the study period, the adult ICUs consisted of 49-beds. Two general ICUs (21 beds), one neurological ICU (8 beds), and two cardiologic ICUs (20 beds). The ICU staffing comprised of a physician (staff-to-patient ratio 1:5), registered nurse (staff-to-patient ratio 1:2), and physiotherapist (staff-to-patient ratio 1:5). A daily multidisciplinary round to determine the goals of care was performed in both periods, considering the ABCDE bundle. The EM goal in the before period was one of the discussion topics among the team during the rounds but without the use of a mobility tool to guide that decision.

### Data collection

Data was retrospectively collected from admission to hospital discharge from medical records and hospital electronic databases. Patient baseline information including demographics, comorbidities, and severity of illness at ICU admission and readmission was obtained from Epimed Solutions^®^.

Using a standardized checklist (Supplementary), the same professional already in charge of the institutional indicator was responsible for the “verticalization” and “non-compliance” rate collection, in order to maintain greater reliability and validity. The “verticalization” and “non-compliance” rate were monitored for a period of four months before and after the QI initiative. The presence of ICU-AW was assessed as well with MRC in patients alert and able to follow commands, as recommended by the institutional mobilization protocol. It was defined by MRC < 48 in patients that had no plausible etiology for weakness other than critical illness^27^. SOMS scale was applied only to patients unable to follow commands, and the presence or absence of this form of evaluation was assessed on both groups.

Variables related to ICU-AW risk factors as mechanical ventilation > 72 h, use of sedation, analgesia, neuromuscular blockers, corticosteroids, sepsis, septic shock, and immobilism (defined as the permanence on bed for more than 50% of the day) were collected.

The project researchers, through our institutional electronic medical records, also collected variables related to the outcomes as ICU length of stay, mobilization barriers and the highest achieved mobility landmark of the day. All mobilization’s barriers perceived by the physical therapists during their care were also recorded; as presence of sedation, patient’s devices (urinary catheter, nasogastric tube, arterial or venous lines), mechanical circulatory assist device, vasoactive drugs, renal supplementation therapy, presence of pain, weakness, endotracheal intubation, tracheostomy, mechanical ventilation, non-invasive ventilation or high flow nasal cannula.

If any patient’s information was missing, the researchers or the professional in charge of the institutional indicator could access the assistance team to resolve relevant queries. The checklist and the spreadsheet used to compute the data were double-checked to avoid any data loss or incomplete data.

### Improvement strategy

To elaborate the improvement strategy, initially, we performed a summary of the evidence considering out-of-bed mobilization. Posteriorly, we optioned to understand the problem from the perspective of the multidisciplinary team. We organized a brainstorming during a workshop freely listed by the participants (the results were grouped in an Ishikawa diagram) and an online multiple-choice questionnaire. Both addressing the modifiable barriers related to environment, patient, staff, and process. Finally, the team perception was paired with the data obtained from a meticulous verification of the patient’s medical records to elucidate modifiable barriers involved in the cases of patients who were moveable and those who were not^28^.

**Figure 2:**
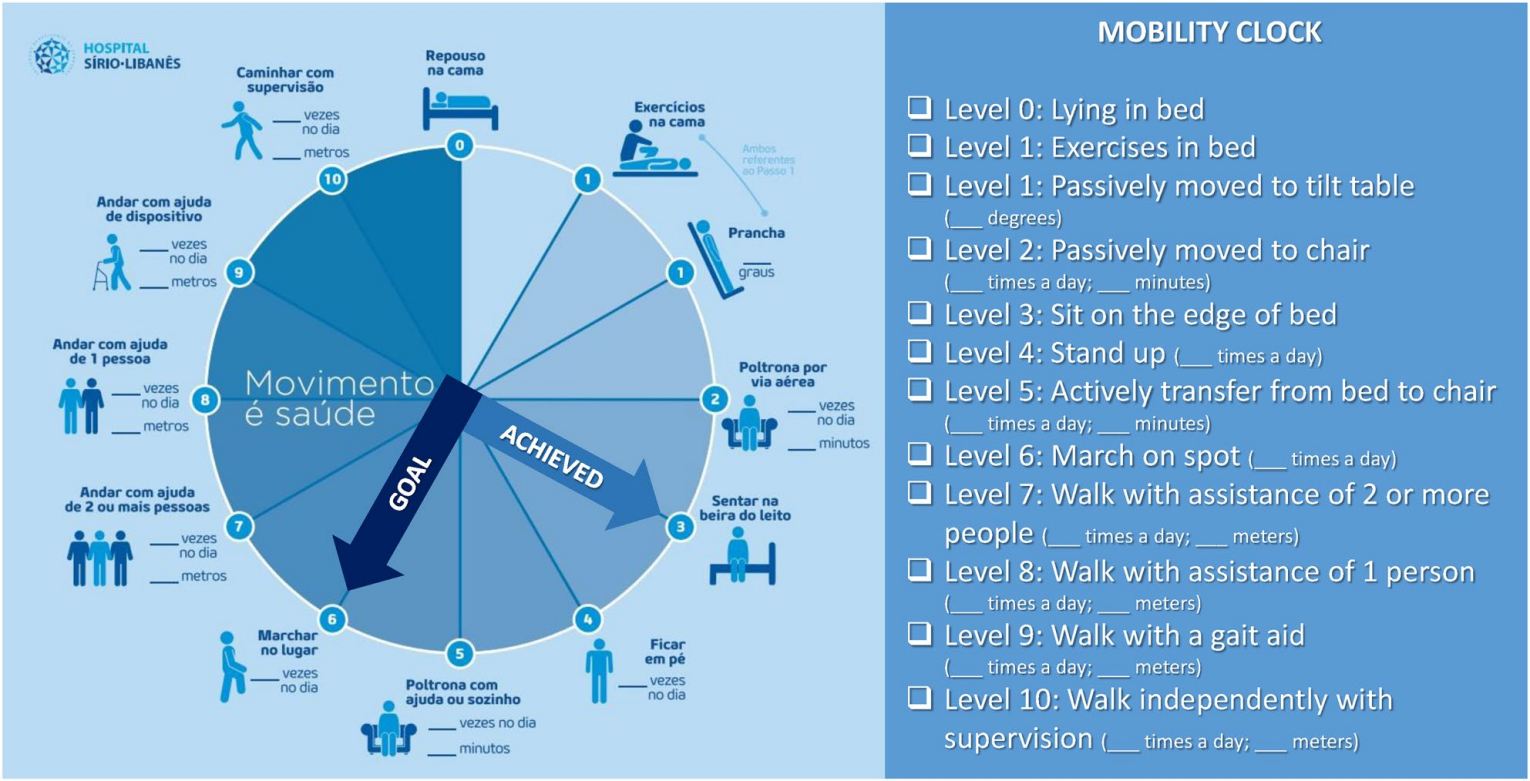
The mobility clock monitors the level of mobility in the intensive care units of Hospital Sírio-Libanês and is based on the ICU mobility scale. It presents ten mobility milestones (the higher the score, the higher the mobility level achieved by the patient). One of the hands of the clock represents the mobility level planned by the multidisciplinary team for the patient during the shift (goal), and the other, represents what was achieved. In the example above, the objective elaborated by the team was to “march on spot” (level 6) and the milestone achieved was to “sit on the edge of bed” (level 3). Thus, the objective was not reached because the level of mobility achieved was lower than planned.

After analyzing the results of these activities, we verified the importance of improving communication between the characters involved, planning, and process individualization considering the specific barriers, in addition to patient and family participation. Considering these points, a visual tool was developed named as “mobility clock” (Fig. 2) to simultaneously quantify, inform, and monitor the patient's functional level. Instead of hours, it displays the different landmarks of mobility based on the ICU mobility scale^29^.

An action plan was prepared and set in motion to inaugurate the “mobility clock” during the “mobilization week”. It consisted in various activities created for the multidisciplinary team regarding the importance of ICU mobilization. To sensitize the team about the importance of EM, the week initiated with a talk show from a patient who developed quadriplegia due to immobilization. The patient, his family members and the professionals involved shared their personal experience during the period of ICU hospitalization and the impact after hospital discharge. During that week, a lecture with updated literature data on out-of-bed ICU mobilization was ministered.

The internal audit results along with institutional modifiable barriers were presented along with the “mobility clock” through an animation specifically produced to explain its development and application. To conclude it, the “mobilization challenge” was released. It consisted in a four-week competition between the ICU units with the purpose of establishing the use of the “mobility clock” by the team. The winning unit would be the one that mobilized the largest number of eligible patients using the “mobility clock”. During this period, a podcast explaining how to use the “mobility clock” and the challenge rules was broadcasted on the institution’s channels. Banners referring to mobility were displayed at the entrance of each unit. To motivate the staff to the cause, stickers encouraging mobilization and chocolate treats were distributed ^28^.

The “mobility clock” was placed in the ICU rooms to be visible to the patient, family, and staff. One clock pointer marked the mobility level that was set as the goal and the other the landmark that the patient achieved. To improve communication in the multidisciplinary round, the expected goal per patient was determined considering the barriers presented, as well as the physical condition of each individual. At this time, if possible, the importance of reaching the chosen mobility landmark on the day was explained to the patient and family. The clock hand that corresponded to the landmark achieved by the patient was moved during the day as soon as the mobility level was reached.

During the before-after period, no other institutional strategy regarding EM was employed.

### Statistical analysis

The sample size (at least 88 patients per period) was calculated based on a previous pilot study considering the number necessary to reduce the “non-compliance” with the protocol on 10%, given a two-tailed type 1 error of 5% and a power of 80%. Quantitative continuous variables were compared using the Mann–Whitney U test for non-normally distributed variables with interquartile values used to represent data dispersion. The means of normally distributed variables were compared using Student’s t-test. Pearson’s chi-squared test and Fisher’s exact test were used to compare the categorical variables. The significance level was set at p ≤ 0.05 (two-tailed).

## Results

**Figure 3:**
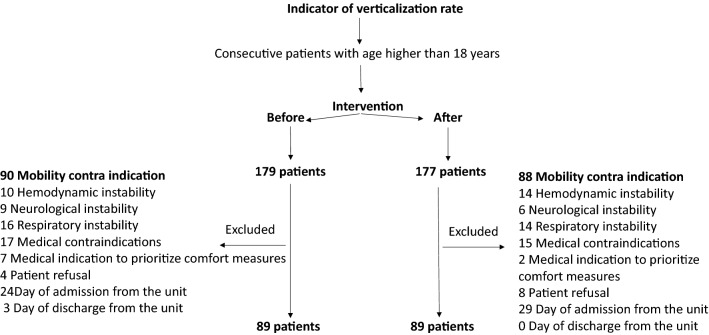
Study sample flowchart.

The EM protocol “verticalization” rate was monitored for a period of four months before and after the strategy to achieve the sample. In the “before” period, 179 patients were screened. Of these, 90 were excluded because they presented at least one mobility contraindication. During the “after” period, 177 patients were screened, with the exclusion of 88 patients, for the same reason as mentioned above, remaining 89 patients on this period (Fig. 3). Both groups had similar baseline characteristics (Table 1).

**Table 1:** Baseline characteristics.

	Before (89 patients)	After (89 patients)	P
**Female, n (%)**	36 (40.45)	31 (34.83)	^a^0.44
**BMI, median (interquartile)**	25.6 (23.1–29.35)	25.9 (23.4–28.4)	^b^0.73
**SAPS-3, mean (± standard deviation)**	46 (13.5)	44.9 (13.5)	^c^0.56
**SOFA, median (interquartile)**	3 (1–5)	3 (1–5)	^b^0.94
**ICU admission reason, n (%)**			^a^0.69
Emergency surgery	6 (6.74)	6 (6.74)	
Elective surgery	22 (24.72)	27 (30.34)	
Clinic	61 (68.54)	56 (62.2)	
**DM, n (%)**	28 (31.46)	32 (35.95)	^a^0.53
**SAH, n (%)**	49 (55.06)	52 (58.43)	^a^0.65
**CKD, n (%)**	15 (16.85)	13 (14.61)	^a^0.68
**HF n (%)**	23 (25.84)	13 (14.61)	^a^0.06
**COPD/asthma, n (%)**	9 (10.11)	11 (12.36)	^a^0.63

*BMI* body mass index, *SAPS 3* Simplified Acute Physiology Score, *SOFA* Sequential Organ Failure Assessment, *ICU* intensive care unit, *DM* diabetes mellitus, *SAH* systemic arterial hypertension, *CKD* chronic kidney disease, *HF* heart failure, *COPD* chronic obstructive pulmonary disease.

^a^Pearson chi-square, ^b^Mann–Whitney, ^c^t student.

The risk factors for ICU-AW presented at indicator data were similar between the groups except for the use of corticosteroids that was significantly greater in the “after” period (29.21% vs. 14.61%, p = 0.02) (Table 2). Regarding the perceived barriers, the need of non-invasive ventilation (6.74% vs 1.12%, p = 0.12) and mechanical ventilation (11.24% vs 4.49%, p = 0.16) was greater in the “after” period; however, no statistical significance was achieved (Table 3).

**Table 2:** Risk factors for ICU-AW.

	**Before (89 patients)**	**After (89 patients)**	**p**
MV > 72 h, n (%)	7 (7.87)	8 (8.99)	^b^1
Sedation > 72 h, n (%)	7 (7.87)	8 (8.99)	^b^1
Analgesia, n (%)	11 (12.36)	19 (21.35)	^a^0.19
NMB, n (%)	2 (2.25)	1 (1.12)	^b^1
Septic shock, n (%)	9 (10.11)	11 (12.36)	^b^0.81
Sepsis, n (%)	20 (22.47)	14 (16.09)	^a^0.28
Corticosteroids, n (%)	13 (14.61)	26 (29.21)	^**a**^**0.02**

Bold values denote statistical significance at the p ≤ 0.05 level.

*MV* mechanical ventilation, *NMB* neuromuscular blocker.

^a^Pearson chi-square, ^b^Fisher.

**Table 3:** Perceived barriers.

	Before (89 patients)	After (89 patients)	p
Sedation	4 (4.49)	8 (8.99)	^b^0.54
Devices	37 (41.57)	48 (53.93)	^a^0.10
MCAD	3 (3.37)	2 (2.25)	^b^1
VADs, n (%)	14 (15.73)	12 (13.48)	^a^0.67
RST	5 (7.87)	6 (6.74)	^b^1
Pain	11 (12.76)	19 (21.35)	^a^0.11
Weakness	25 (28.09)	23 (25.84)	^a^0.74
ETT, n (%)	2 (2.25)	5 (5.52)	^b^0.44
Tracheostomy, n (%)	9 (10.11)	7 (7.87)	^b^0.79
MV, n (%)	4 (4.49)	10 (11.24)	^b^0.16
NIV, n (%)	1 (1.12)	6 (6.74)	^b^0.12
HFNC, n (%)	3 (3.37)	3 (3.37)	^a^1

*MCAD* mechanical circulatory assist device, *VADs* vasoactive drugs, *RST* renal supplementation therapy, *ETT* endotracheal intubation, *MV* mechanical ventilation, *NIV* non-invasive ventilation, *HFNC* high flow nasal cannula.

^a^Pearson chi-square, ^b^Fisher.

The ICU-AW identified by means of an MRC < 48 was similar between the groups. The proportion of patients whose MRC was not possible to be applied, requiring SOMS to be performed, was higher in the “after” period but without statistical significance (Table 4).

**Table 4:** ICU-AW.

	Before (89 patients)	After (89 patients)	p
**MRC, n (%)**			
0–23	8 (8.99)	1 (1.12)	^a^0.06
24–35	4 (4.49)	3 (3.37)	
37–47	18 (20.22)	12 (13.48)	
48–60	45 (50.56)	50 (56.18)	
**MRC < 48, n (%)**	44 (49.44)	39 (43.82)	^a^0.45
**SOMS**	14 (15.73)	23 (25.84)	^b^0.13

*MRC* Medical Research Council Scale, *SOMS* Surgical Intensive Care Unit Optimal Mobilisation Score.

^a^Pearson chi-square, ^b^Fisher.

**Figure 4:**
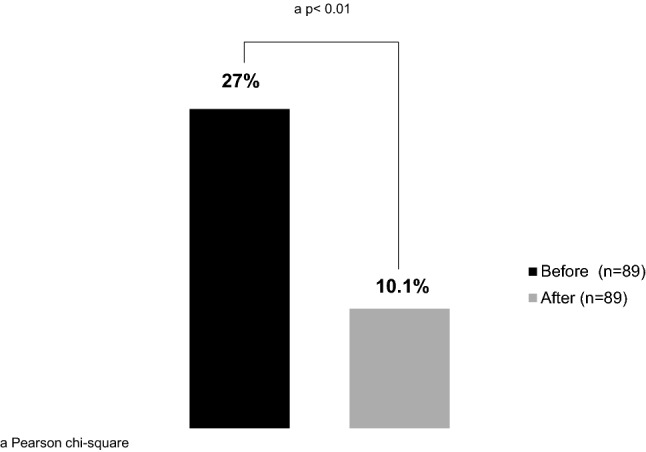
Institutional protocol “non-compliance” rate.

**Figure 5:**
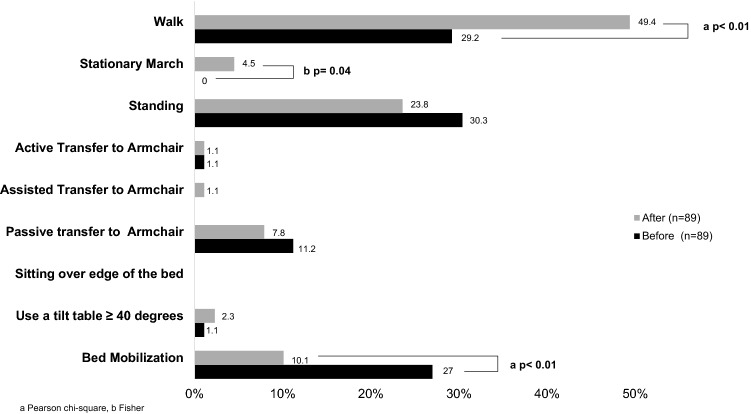
Proportion of highest mobility landmark achieved.

After the QI, a lower “non-compliance” rate with the protocol was observed, compared to the previous period (10.11% vs. 26.97%, p < 0.004) (Fig. 4). Considering the mobility landmark, the proportion of patients walking was higher in the “after” period compared to the “before” period (49.44% vs. 29.21%, p = 0.006), as well marching on spot that was performed in 4.49% of the patients on the “after” period while it was not performed on the period before QI (p = 0.04) (Fig. 5).

No differences between the periods were observed in the hospital and lCU length of stay, as well as in the mortality rate. However, the ICU readmission rate was lower on the “after” period (2.25% vs. 11.24%, p = 0.017) (Table 5).

**Table 5:** Hospital and ICU length of stay, hospital and ICU death and readmission rate.

	Before (89 patients)	After (89 patients)	p
ICU days, median (interquartile)	4 (2–10.50)	3 (2–11)	^b^0.53
Hospital days, median (interquartile)	15.5 (7.75–32)	15 (7–34)	^b^0.91
Hospital mortality, n (%)	11 (12.79)	7 (8.64)	^a^0.38
ICU mortality, n (%)	4 (4.49)	3 (3.37)	^a^0.70
ICU readmission, n (%)	10 (11.24)	2 (2.25)	^**a**^**0.017**

Bold values denote statistical significance at the p ≤ 0.05 level.

^a^Pearson Chi-Square, ^b^Mann–Whitney.

## Discussion

The non-compliance rate reduction with the QI strategy, suggests an effectiveness in the short term to achieve our goals, in accordance with results from previous QI studies^18–20^. These, with the increase observed in highest level of mobility landmark archived in after period, may be a consequence of the QI strategies, that provided an individualized discussion per patient during the definitions of multidisciplinary goals. That practice along enhanced the adherence and performance of the multidisciplinary team in addition to improving the communication.

ICU EM is essential to prevent a loss of functional status that can perpetuate after hospital discharge, affecting the quality of life and social reintegration^1–7^. Besides considered feasible in cases of a favorable clinical condition, it is frequent that not out of bed mobilization occur due to modifiable barriers. In this way, “non-compliance” with EM protocols can be secondary to failure to identify institutional barriers as well as failure to develop strategies to overcome it^12,14,24^.

The “non-compliance” rate indicator was used to verify the effectiveness of our strategy as the last stage foreseen in the QI process^30^. Except for the proposed strategy aimed at EM, in the after period there was no difference between the units to the other items proposed in the evidence-based guide ABCDE bundle.

The mobility clock was created after understanding the problem of our institution with the barriers not only perceived by the professionals in clinical practice, but also those verified through an active search for specific cases of “non-compliance” with our protocol. After its development, strategies to sensitize and educate healthcare professionals, patients, and family members to prioritize out-of-bed mobilization were developed as well an execute strategic plan was done as recommended in literature^8,18,20^.

The visualization of the mobilization objective of the day, moreover, to continually demonstrating to the team the landmark to be reached on the day, may have led the patient and/or family members to perceive that out-of-bed mobilization in addition to being important, is feasible and safety in the ICU. Making them partner in the process.

It is known that the family participation throughout the rehabilitation process, as recommended most recently with the inclusion of the item “F” in the ICU bundle, can optimize patient recovery^2,7^. In this QI project, the purpose of the “mobility clock” was explained to the family and, although we have noticed their greater involvement in achieving higher mobility levels by their relatives, no family-related outcomes were measured.

During the monitored periods the results were similar regarding the risk factors for ICU-AW and perceived barriers.

In the post-strategy period, a decrease in readmission rate was observed. It is important to note that sample size of this study was not calculated to investigate this primary purpose. Certainly, it is an important data to be confirmed in further studies since after a long-term ICU stay (> 72 h), one year mortality was approximately 28% and was related to age, disease severity, comorbidities, and ICU re-admissions^31^.

A limitation of our study was the before-and-after design that carries the biases inherent to the temporal variation and the impact of confounders that were not considered, as well as the single-center QI limiting the external validity. Further research on this topic should be implemented, including multicenter prospective randomized studies focusing on the clinical and economic outcomes of mobilization QI projects.

## Conclusion

The multifaceted strategy was effective to reduce “non-compliance” rate to EM protocol of a tertiary hospital. In addition, promoted a higher level of mobility landmark. In further research, it would be interesting to study the effectiveness of these strategies in different centers, with more suitable study designs involving clinical outcomes as the readmission rate. Furthermore, considering hospital and ICU length of stay, the economic impact of these mobilization-QI projects is also an important field of study and may encourage future investigations.

A QI multidisciplinary approach in a brief period can positively influence patient mobilization and team engagement without additional costs for the service and with a simple and intuitive tool.

## Supplementary Information

Supplementary Information.

## Data Availability

All data generated or analyzed during this study are included in this published article [and its supplementary information files].

## Abbreviations

ICU: Intensive care unit
MRC: Medical Research Council
SOMS: Surgical Intensive Care Unit Optimal Mobilization Score
ICU-AW: Intensive care unit acquired weakness
BMI: Body mass index
SAPS 3: Simplified Acute Physiology Score
SOFA: Sequential Organ Failure Assessment
DM: Diabetes mellitus
SAH: Systemic arterial hypertension
CKD: Chronic kidney disease
HF: Heart failure
COPD: Chronic obstructive pulmonary disease
MV: Mechanical ventilation
NMB: Neuromuscular blocker
MCAD: Mechanical circulatory assist device
VADs: Vasoactive drugs
RST: Renal supplementation therapy
ETT: Endotracheal intubation
NIV: Non-invasive ventilation
HFNC: High flow nasal cannula.
QI: Quality improvement
