# Supplementary material for: Effectiveness of a quality improvement strategy with implementation of a specific visual tool to promote ICU early mobilization
Source: Sci Rep. 2022 Oct 13;12:17206. doi: 10.1038/s41598-022-21227-y (PMC9562414; doi:10.1038/s41598-022-21227-y)
Supplement: Supplementary file 1 — Supplementary Information. [file 41598_2022_21227_MOESM1_ESM.docx]

**Online Data Supplement**

**Effectiveness of a quality improvement strategy with implementation of a specific visual tool to promote ICU early mobilization**

Patricia Nery de Souza^1^, Jessica Borges Kroth^1^, Amanda dos Santos Ligero^1^, Juliana Mesti Mendes^1^, Ana Lígia Vasconcelos Maida^1^, Laerte Pastore^1^ and Wellington Pereira dos Santos Yamaguti^1^.

^1^Hospital Sírio Libanês, São Paulo, Brazil.

**Index**

1. Collection Checklist

page:1

1. Revised Standards for Quality Improvement Reporting Excellence (SQUIRE 2.0)

page:4

| COLLECTION CHECKLIST | | | | | | |
| --- | --- | --- | --- | --- | --- | --- |
| 1.PATIENT AND ICU DATA | | | | | | |
| Name: | | | **ICU wing:**  1. [ ] I 2. [ ] II 3. [ ] IV 4. [ ] UAIC 5. [ ] Cardio | | | |
| Collection day: _____/_____/______ | | | **Chart number:** _______________­­­­­____________  **Patient ID number:** _______________­­­­­__________ | | | |
| 2. SOCIODEMOGRAPHIC DATA | | | | | | |
| Sexo: 1. [ ] Female 2. [ ] Male | | | **Age:**  _______________ | | | |
| 3. ANTROPHOMETRY | | | | | | |
| Weight and height:  1. _________ 2. _________ | | | **BMI:**  1. [ ] Underweight 2. [ ] Normal 3. [ ] Overweight 4. [ ] Obese | | | |
| 4. HOSPITAL AND ICU LENGHT OF STAY | | | | | | |
| Hospital lenght of stay: __________ | | | **ICU lenght of stay: ___________** | | | |
| 5. PREVIOUS INFORMATION | | | | | | |
| Previous mobility status:  1. [ ] Able to get out of bed 2. [ ] Not able to get out of bed 3. [ ] Not informed | | | **Chronic diseases:**  1. [ ] Diabetes 2. [ ] Hipertension 3. [ ] Chronic kidney disease 4. [ ] Heart failure 5. [ ] Asthma/COPD 6. [ ] Dyslipidemia 7. Other________________ | | | |
| Causa da admissão: 1. [ ] Clínico 2. [ ] Cirúrgico 3. [ ] Clínico cirúrgico  ________________________________________________________________________________ | | | | | | |
| 6. NEUROLOGICAL ASSESSMENT | | | | | | |
| RASS:  1. [ ] Deep sedated (-4 to -5)  2. [ ] Moderated sedated (-2 to -3)  3. [ ] Light sedated (-1 to +1)  4. [ ] Agitated (+2 to +4)  5. [ ] Not informed | ***Glasgow coma Scale*:**  1. [ ] Deep coma (3 to 8)  2. [ ] Moderate coma (9 to 12)  3. [ ] Mild coma (13 to 15)  4. [ ] Normal (15)  5. [ ] Not informed | | | | | **CAM-ICU*:***  1. [ ] CAM ICU +  2. [ ] CAM ICU -  3. [ ] Not informed |
| 7. Organic Dysfunction | | | | | | |
| SOFA Score: _______  [ ] None | | **Sepsis:**  1. [ ] Yes 2. [ ] No 3. [ ] Not informed | | | | **Septic Shock:**  1. [ ] Yes 2. [ ] No 3. [ ] Not informed |
| 8. FUNCTIONAL PLAN | | | | | | |
| MRC Score: ________  1. [ ] Severe weakness (0-23)  2. [ ] Moderate weakness (24-35)  3. [ ] Mild weakness (36-47)  4. [ ] Risk of weakness (48-60)  6. [ ] Not informed | | | | **SOMS: ________**  1. [ ] No mobility  2. [ ] Able to sit  3. [ ] Able to stand  4. [ ] Able to deambulate  5. [ ] Not informed | | |
| 9. PATIENT’S GRAVITY | | | | | | |
| APACHE II ____________ | | | | | **SAPS3** ____________ | |
| 10. VERTICALIZATION AND OUT OF BED MOBILIZATION | | | | | | |
| Maximum functional status achieved?  1.[ ] Yes 2. [ ] No | | | | **Verticalization occurred?**  1.[ ] Yes 2. [ ] No | | |
| Clinical conditions to be verticalized?  1.[ ] Yes 2. [ ] No | | | | | | |
| Reason not to verticalize was documented?  1.[ ] Yes 2. [ ] No | | | | | | |
| If YES: What was the most important non-modifiable barrier?  1. [ ] Medical recommendation  2. [ ] Hemodynamic instability  3. [ ] Respiratory instability  4. [ ] Neurological instability  5. [ ] Orthopedic contraindication  6. [ ] Patient’s refusal  7. [ ] Imediate post extubation  8. [ ] None | | | | **What was the most importante modifiable barrier?**  1. [ ] Level of consciousness  2. [ ] Excessive sedation  3. [ ] Vascular accesses and invasive devices  4. [ ] Ventricular assist devices  5. [ ] Renal replacement therapy  6. [ ] Pain  7. [ ] Fatigue  8. [ ] Orotracheal intubation  9. [ ] Vasoactive drugs  10. [ ] Muscle Weakness  11. [ ] None | | |
| What are the mobility milestones reached by the patient?  1. [ ] Tilt table ≥ 40º ____ 2 . [ ] Sitting on the edge of the bed 3. [ ] Passive transfer to armchair  4. [ ] Active armchair transfer 5 . [ ] Sitting at the armchair. 6. [ ] Standing 7. [ ] Marching on spot 8. [ ] Walking | | | | | | |
| What is the highest level of mobility achieved by the patient?  1. [ ] Tilt table ≥ 40º ____ 2 . [ ] Sitting on the edge of the bed 3. [ ] Passive transfer to armchair  4. [ ] Active armchair transfer 5 . [ ] Sitting at the armchair. 6. [ ] Standing 7. [ ] Marching on spot 8. [ ] Walking | | | | | | |
| 11. VENTILATORY STATUS | | | | | | |
| During mobilization patient have:  1. [ ] Orotracheal intubation  2. [ ] Tracheostomy  3. [ ] Mechanical Ventilation  4. [ ] Non-invasive ventilation  5. [ ] High Flow Nasal Cannula Therapy  6. [ ] Oxigenoterapy/Nebulization | | | | | | |
| 12. MUSCLE WEAKNESS RISK FACTORS | | | | | | |
| Are there risk factors for the development of AW-ICU?  1. [ ] Mechanical ventilation > 72 hours 2. [ ] Vasoactive drugs 3. [ ] Sedation 4. [ ] Analgesia 5. [ ] Neuromuscular blocker 6. [ ] Corticosteroid 7. [ ] Immobility | | | | | | |
| Bloco 13. INVASIVE DEVICES | | | | | | |
| Are there vascular accesses/invasive devices?  1. [ ] Central femoral catheter 2. [ ] Central venous cateter in other place 3. [ ] Hemodialysis with femoral acess 4. [ ] Hemodialysis with other acess  5. [ ] Continuous hemodialysis 6. [ ] Intermittent hemodialysis 7. [ ] femoral arterial cateter 8. [ ]other arterial catheter 9. [ ] Chest tube  10. [ ] Abdominal tube 11. [ ] Indwelling Urinary Catheter 12. [ ] Peripherally inserted central catheter 13. [ ] Other place tube | | | | | | |
| Bloco 14. MECHANICAL CIRCULATORY ASSIST DEVICES | | | | | | |
| Are there any mechanical circulatory assist device present?  1. [ ] Intra-aortic balloon pump 2. [ ] Extracorporeal membrane oxygenation 3. [ ] Centrimag® 4. [ ] Heart Mate® 5. 1. [ ] Other _______ | | | | | | |
| COPD: Chronic Obstructive Pulmonary Disease. RASS: Richmond Agitation-Sedation Scale. CAM-ICU: Confusion Assessment Method in Intensive Care Unit. SOFA: Sequential Organ Failure Assessment. MRC: Medical Research Council SOMS: Surgical Intensive Care Unit Optimal Mobilisation Score. APACHE II: Acute Physiology and Chronic Health Evaluation II. SAPS 3: Simplified Acute Physiology Score 3. | | | | | | |

**2. Revised Standards for Quality Improvement Reporting Excellence (SQUIRE 2.0)**

| **Text Section and Item**  **Name** | **Section or Item Description** |
| --- | --- |
| **Notes to authors** | - The SQUIRE guidelines provide a framework for reporting new   knowledge about how to improve healthcare   - The SQUIRE guidelines are intended for reports that describe [system](#_bookmark13) level work to improve the quality, safety, and value of healthcare, and used methods to establish that observed outcomes were due to the [intervention(s).](#_bookmark8) - A range of approaches exists for improving healthcare. SQUIRE may be adapted for reporting any of these. - Authors should consider every SQUIRE item, but it may be inappropriate or unnecessary to include every SQUIRE element in a particular manuscript. - The SQUIRE Glossary contains definitions of many of the key words in SQUIRE. - The Explanation and Elaboration document provides specific examples of well-written SQUIRE items, and an in-depth explanation of each item. - Please cite SQUIRE when it is used to write a manuscript. |
| **Title and Abstract** |  |
| **1. Title** | Indicate that the manuscript concerns an [initiative](#_bookmark6) to improve healthcare (broadly defined to include the quality, safety, effectiveness, patient- centeredness, timeliness, cost, efficiency, and equity of healthcare). ✓ |
| **2. Abstract** | 1. Provide adequate information to aid in searching and indexing 2. Summarize all key information from various sections of the text using the abstract format of the intended publication or a structured summary such as: background, local [problem,](#_bookmark10) methods, interventions,   results, conclusions ✓ |
| **Introduction** | *Why did you start?* |
| [**3. Problem**](#_bookmark10) [**Description**](#_bookmark10) | Nature and significance of the local [problem](#_bookmark10).  Lines 37- 63 |
| **4. Available knowledge** | Summary of what is currently known about the [problem,](#_bookmark10) including relevant previous studies.  Lines 30-44 |
| **5.** [**Rationale**](#_bookmark12) | Informal or formal frameworks, models, concepts, and/or [theories](#_bookmark14) used to explain the [problem,](#_bookmark10) any reasons or [assumptions](#_bookmark0) that were used to develop the [intervention(s),](#_bookmark8) and reasons why the [intervention(s)](#_bookmark8) was  expected to work.  Lines 45-63 |
| **6. Specific aims** | Purpose of the project and of this report Lines 65-67 |
| **Methods** | *What did you do?* |
| **7.** [**Context**](#_bookmark1) | Contextual elements considered important at the outset of introducing the [intervention(s)](#_bookmark8)  Lines 134-144 |
| **8.** [**Intervention(s)**](#_bookmark8) | 1. Description of the [intervention(s)](#_bookmark8) in sufficient detail that others could reproduce it 2. Specifics of the team involved in the work   Lines 131-168 |
| **9. Study of the Intervention(s)** | 1. Approach chosen for assessing the impact of the [intervention(s)](#_bookmark8) 2. Approach used to establish whether the observed outcomes were due to the [intervention(s)](#_bookmark8) 3. Lines 79-94 |
| **10. Measures** | 1. Measures chosen for studying [processes](#_bookmark11) and outcomes of the [intervention(s),](#_bookmark8) including rationale for choosing them, their operational definitions, and their validity and reliability 2. Description of the approach to the ongoing assessment of contextual elements that contributed to the success, failure, efficiency, and cost 3. Methods employed for assessing completeness and accuracy of data   Lines 82-94 and 105-129 |
| **11. Analysis** | 1. Qualitative and quantitative methods used to draw [inferences](#_bookmark5) from the data 2. Methods for understanding variation within the data, including the   effects of time as a variable  Lines 177-183 |
| **12. Ethical**  **Considerations** | [Ethical aspects](#_bookmark2) of implementing and studying the [intervention(s)](#_bookmark8) and how they were addressed, including, but not limited to, formal ethics review and potential conflict(s) of interest  Lines 71-77 |
| **Results** | *What did you find?* |
| **13. Results** | 1. Initial steps of the [intervention(s)](#_bookmark8) and their evolution over time (*e.g.*, time-line diagram, flow chart, or table), including modifications made to the intervention during the project 2. Details of the [process](#_bookmark11) measures and outcome 3. Contextual elements that interacted with the [intervention(s)](#_bookmark8) 4. Observed associations between outcomes, interventions, and relevant contextual elements 5. Unintended consequences such as unexpected benefits, problems, failures, or costs associated with the [intervention(s).](#_bookmark8) 6. Details about missing data   Lines 185-210 |
| **Discussion** | *What does it mean?* |
| **14. Summary** | 1. Key findings, including relevance to the [rationale](#_bookmark12) and specific aims 2. Particular strengths of the project   Lines 213-254 |

| **15. Interpretation** | 1. Nature of the association between the [intervention(s)](#_bookmark8) and the outcomes 2. Comparison of results with findings from other publications 3. Impact of the project on people and [systems](#_bookmark13) 4. Reasons for any differences between observed and anticipated outcomes, including the influence of [context](#_bookmark1) 5. Costs and strategic trade-offs, including [opportunity costs](#_bookmark9)   Lines 219-249 |
| --- | --- |
| **16. Limitations** | 1. Limits to the [generalizability](#_bookmark3) of the work 2. Factors that might have limited [internal validity](#_bookmark7) such as confounding, bias, or imprecision in the design, methods, measurement, or analysis 3. Efforts made to minimize and adjust for limitations   Lines 250-254 |
| **17. Conclusions** | 1. Usefulness of the work 2. Sustainability 3. Potential for spread to other [contexts](#_bookmark1) 4. Implications for practice and for further study in the field 5. Suggested next steps   Lines 257-264 |
| **Other information** |  |
| **18. Funding** | Sources of funding that supported this work. Role, if any, of the funding organization in the design, implementation, interpretation, and reporting  Line 309 |

Table 2. Glossary of key terms used in SQUIRE 2.0. This Glossary provides the intended meaning of selected words and phrases as they are used in the SQUIRE 2.0 Guidelines. They may, and often do, have different meanings in other disciplines, situations, and settings .

Assumptions

Reasons for choosing the activities and tools used to bring about changes in healthcare services at the system level.

Context

Physical and sociocultural makeup of the local environment (for example, external environmental factors, organizational dynamics, collaboration, resources, leadership, and the like), and the interpretation of these factors (“sense-making”) by the healthcare delivery professionals, patients, and caregivers that can affect the effectiveness and generalizability of intervention(s).

Ethical aspects

The value of system-level initiatives relative to their potential for harm, burden, and cost to the stakeholders. Potential harms particularly associated with efforts to improve the quality, safety, and value of healthcare services include opportunity costs, invasion of privacy, and staff distress resulting from disclosure of poor performance.

Generalizability

The likelihood that the intervention(s) in a particular report would produce similar results in other settings, situations, or environments (also referred to as external validity).

Healthcare improvement

Any systematic effort intended to raise the quality, safety, and value of healthcare services, usually done at the system level. We encourage the use of this phrase rather than “quality improvement,” which often refers to more narrowly defined approaches.

Inferences

The meaning of findings or data, as interpreted by the stakeholders in healthcare services – improvers, healthcare delivery professionals, and/or patients and families

Initiative

A broad term that can refer to organization-wide programs, narrowly focused projects, or the details of specific interventions (for example, planning, execution, and assessment)

Internal validity

Demonstrable, credible evidence for efficacy (meaningful impact or change) resulting from introduction of a specific intervention into a particular healthcare system.

Intervention(s)

The specific activities and tools introduced into a healthcare system with the aim of changing its performance for the better. Complete description of an intervention includes its inputs, internal activities, and outputs (in the form of a logic model, for example), and the mechanism(s) by which these components are expected to produce changes in a system’s performance.

Opportunity costs

Loss of the ability to perform other tasks or meet other responsibilities resulting from the diversion of resources needed to introduce, test, or sustain a particular improvement initiative

Problem

Meaningful disruption, failure, inadequacy, distress, confusion or other dysfunction in a healthcare service delivery system that adversely affects patients, staff, or the system as a whole, or that prevents care from reaching its full potential

Process

The routines and other activities through which healthcare services are delivered

Rationale

Explanation of why particular intervention(s) were chosen and why it was expected to work, be sustainable, and be replicable elsewhere.

Systems

The interrelated structures, people, processes, and activities that together create healthcare services for and with individual patients and populations. For example, systems exist from the personal self- care system of a patient to the individual provider-patient dyad system, to the microsystem, to the macrosystem, and all the way to the market/social/insurance system. These levels are nested within each other.

Theory or theories

Any “reason-giving” account that asserts causal relationships between variables (causal theory) or that makes sense of an otherwise obscure process or situation (explanatory theory). Theories come in many forms and serve different purposes in the phases of improvement work. It is important to be explicit and well-founded about any informal and formal theory (or theories) that are used.
